# In Vitro and In Vivo Antioxidant Activity of *Agave sisalana* Agro-Industrial Residue

**DOI:** 10.3390/biom10101435

**Published:** 2020-10-12

**Authors:** Stella Maria Andrade Gomes Barreto, Cesar Orlando Muñoz Cadavid, Rafael Amir de Oliveira Moura, Giovanna Melo Martins Silva, Samara Vitória Ferreira de Araújo, Jean Antônio Aderaldo da Silva Filho, Hugo Alexandre Oliveira Rocha, Riva de Paula Oliveira, Raquel Brandt Giordani, Márcio Ferrari

**Affiliations:** College of Pharmacy, Federal University of Rio Grande do Norte, Natal 59012-570, Brazil; smagbarreto@gmail.com (S.M.A.G.B.); cmunozcadavid@gmail.com (C.O.M.C.); amirrafaeloliv@gmail.com (R.A.d.O.M.); giomartins_@hotmail.com (G.M.M.S.); samaravitoria1@gmail.com (S.V.F.d.A.); jeanantoniofilho@gmail.com (J.A.A.d.S.F.); hugo@cb.ufrn.br (H.A.O.R.); rivapoliveira@gmail.com (R.d.P.O.); raquebg@hotmail.com (R.B.G.)

**Keywords:** *Agave sisalana*, agro-industrial, antioxidant, residue

## Abstract

*Agave sisalana* agro-industrial residue has considerable potential against damage associated with oxidative stress and skin aging. This study aims to demonstrate, in vitro and in vivo, the potential of *Agave sisalana* agro-industrial residue as a safe and effective alternative for the prevention of damage caused by oxidative stress and aging. The antioxidant activity was evaluated in vitro (total antioxidant capacity, reducing power, DPPH radical scavenging, metal chelating (Fe^2+^ and Cu^2+^), and hydroxyl radical scavenging) and in vivo using the *Caenorhabditis elegans* organism model. The extract showed in vitro antioxidant activity in all tests performed. Tests with C. *elegans* showed that the extract was able to reduce the intracellular levels of reactive oxygen species (ROS) and increase the survival rate of worms. A downregulation of *gst-4::GFP* expression suggests a direct action against free radicals. *Agave sisalana* agro-industrial residue extract (AsRE) can therefore be considered as a source of antioxidant biomolecules, and the use of this agro-industrial residue in a new production process can lead to sustainability and socioeconomic development.

## 1. Introduction

*Agave* belongs to the family Agavaceae and is widely distributed in tropical and subtropical regions of the world, and, due to their ability to grow in dry lands and their several potential applications, plants of this genus have been called “plants of the century” [1].

The species *Agave sisalana*, commonly known as sisal, is a species that originates from Mexico and is widely cultivated in the Northeast region of Brazil [2]. This species presents a great socioeconomic importance for these regions because it is the main source of hard fiber worldwide, and is used for the production of threads and ropes [3] that are used in the agriculture and marine industries due to their resistance and durability [4,5]. Furthermore, the cultivation of *A. sisalana* has a significant socioeconomic role, especially for families that live around the productive chain. Those families have their jobs and main incomes based on crop management, crop harvesting, crop defibration, fiber processing, industrialization and/or handicrafts production [6].

Approximately 96.0% of the gross weight of *A. sisalana* leaves used to obtain fibers is discarded as agro-industrial residue with no commercial value and prospectively can cause environmental damage [3,4]. However, this residue is reported as being a promising source of biomolecules with potential for food, cosmetic and pharmaceutical applications. Among these biomolecules, the most commonly studied are saponins [1] and phenolic compounds [7]. The main saponins found in *Agave* species are sapogenins [3,8,9] and saponins linked to glycosidic structures [3]. Furthermore, these species also synthesize flavonoids, homoisoflavonoids, and phenolic acids as the main phenolic compounds [7]. In addition, it is important to highlight that agro-industrial *A. sisalana* residue has a high content of sugars (~65%) [10], these being mainly pectins [4], and heteropolysaccharides containing galactose, glucose, mannose, and rhamnose [5,11], and, as well as these, arabinose and ribose were also found [11].

Oxidative stress (OS) results from an imbalance between the production of reactive species and their degradation, which is associated with different physiological disorders [12]. In addition, OS causes cumulative damage in molecules (proteins, lipids, DNA, RNA, carbohydrates) in the human body, which accelerates aging [13].

Therefore, antioxidant biomolecules can act against the process involving OS by fighting or preventing its damage due to their antioxidant activity. These antioxidant biomolecules can act as an intracellular modulator of OS, acting either directly with reactive species, through the transfer of hydrogen atoms, electron transfer and/or chelation of transition metals [14], or indirectly through the activation of the phase II detoxifiers, which are the major line of defense against oxidative stress and its damage [15]. Based on this rationale, different studies have been carried out aiming to evaluate the intrinsic potential of extracts and biomolecules as potential antioxidants through in vitro spectrophotometric assays and in vivo models such as *C*. *elegans* [16,17,18].

The nematode *C. elegans* is used to investigate the processes involving aging and resistance to oxidative stress, and is preferred because of its short life cycle, rapid reproduction, easy handling and relatively clear mechanism of life extension. *C. elegans* is the model organism most used in research for these purposes as it is an alternative that demonstrates rapid safety and efficacy in the screening of drugs and biomolecules [17,18,19].

Previous studies using polysaccharides [5,11] from the residue of *A. sisalana* showed antioxidant activity in vitro. However, the process to obtain these molecules involves several steps, which makes it expensive. In addition, this is not an ecofriendly process, although extracts are bioactive agents and can be obtained using an ecofriendly process.

Data about in vitro and in vivo safety from residue of *A. sisalana* extract has been presented in previous articles. Araldi et al. [20] evaluated the cytotoxic activity and genotoxic/clastogenic potential in Vero cells, human lymphocytes, and mice. These authors showed that several extracts from *A. sisalana* did not present genotoxicity in Vero cells and mice. Therefore, *A. sisalana* extracts can lead to DNA breaks in human lymphocytes but did not promote DNA damage in mice bone marrow. However, a previous study from our own group showed that hydroethanolic extract from residue of *A. sisalana* did not affect the mammals’ cell viability. Accordingly, in a primary irritability test, it was dropped on the healthy skin of volunteers (men and women, aged between 22 and 46 years old) and after 72 h, the authors did not find signs of irritability on the skin of volunteers [10].

Therefore, given the growing demand for safe and effective compounds and biomolecules for oral and topical use, this study aims to demonstrate, in vitro and in vivo, the potential of *A. sisalana* agro-industrial residue as a safe and effective alternative to prevent aging and oxidative stress.

## 2. Materials and Methods

### 2.1. Materials

Aluminum chloride, quercetin, vanillin, diosgenin, ascorbic acid, 1,1-Diphenyl-2-picrylhydrazyl (DPPH), 2,7-diclorodihydrofluorescein diacetate (H_2_DCFDA), butylated hydroxytoluene (BHT), tert-butylhydroperoxide (TBHP), trichloroacetic acid (TCA), ferrozine, and potassium ferricyanide, were purchased from Sigma-Aldrich (St. Louis, MO, USA). The following were also purchased for use: (Ethylenedinitrilo) tetraacetic acid (EDTA) (Neon, São Paulo, SP, Brazil), ferric chloride (Merk, Darmstadt, Hesse, Germany), sulfuric acid (Labsynth, São Paulo, SP, Brazil), sodium phosphate (Quimiobras, Rio de Janeiro, RJ, Brazil), ammonium molybdate (Vetec, Jaragua do Sul, SC, Brazil), Luria Bertani Medium (Kasvi, São José do Pinhais, PR, Brazil), yeast fermente (Kasvi, São José do Pinhais, PR, Brazil), Nematode Growth Medium (NGM): bacteriological agar and pepton (Kasvi, São José do Pinhais, PR, Brazil), cholesterol (Vetec, Jaragua do Sul, SC, Brazil), calcium chloride (Synth, Diadema, SP, Brazil), magnesium sulfate (Sigma-Aldrich, São Paulo, SP, Brazil), and sodium chloride (Sigma-Aldrich, St. Louis, MO, USA).

### 2.2. Plant Material

Sisaltec Industry (Natal, RN, Brazil) supplied the sisal residue that was collected from a farm directly from a decortication machine in the city of Pedra Grande, Rio Grande do Norte State, Brazil (5°07′56.3″ S 35°54′31.7″ W). The plant material was identified by a botanist (Msc. Alan de Araújo Roque) and the voucher specimen was deposited at the Herbarium of the Federal University of Rio Grande do Norte (UFRN) under number #20544.

### 2.3. Extract Production

The natural fiber (sisal) was mechanically removed from the *A. sisalana* leaves and the residual material from this process (residue) was pressed, providing a liquid portion. The liquid portion was added to ethanol 95 °GL at a 1:3 (*v*/*v*) residue/solvent ratio and stored at 4 °C for 24 h [10]. Subsequently, the liquid extract was filtrated, concentrated, and lyophilized (Christ Alpha 1–2 LD, Osterode am Harz, Germany). The *A. sisalana* agro-industrial residue extract obtained was named AsRE. The yield was calculated from the initial amount of liquid portion according to Equation (1).

Yield (%) = (mass of lyophilized sample/mass of liquid portion) × 100(1)

### 2.4. Chemical Characterization

#### 2.4.1. Total Flavonoids

Total flavonoid was determined by the aluminum chloride coloration method [21]. Briefly, 1.0 mL of AsRE (1.0 mg/mL) was added to 1.0 mL aluminum chloride solution (2.5% *w*/*v*). The reaction mixture was incubated at room temperature, for 60 min, in the dark. Absorbance was measured at 425 nm using a spectrophotometer (Biospectro SP-220, Curitiba, Brazil). The total flavonoids were calculated with a standard curve made with the quercetin standard as a reference (2.0, 5.0, 10.0, 15.0, 20.0, 25.0 and 30.0 µg/mL). The assay was realized in triplicate and the results expressed as µg Eq quercetin (EQ)/mg of AsRE.

#### 2.4.2. Total Saponins

The concentration of total saponins was determined by vanillinsulfuric acid method as described by Ribeiro et al. [22]. Thus, 250 µL of sample (10.0 mg/mL diluted in methanol 80% *w*/*v*) was added to 250 µL of ethanolic solution of vanillin 8% (*w*/*v*) and 2.5 mL of sulfuric acid 72% (*v*/*v*). The resulting acid solution was kept at 60 °C for 10 min. Finally, the absorbance at 544 nm was registered using a spectrophotometer (Biospectro SP-220, Curitiba, Brazil). The standard curve was made using a stock solution of diosgenin (0.50 g/L). The assay was realized in triplicate and the results expressed as percentage.

#### 2.4.3. Fourier Transform InfraRed (FTIR) Spectroscopy

The FTIR was recorded in an IR Prestige-21 spectrophotometer (Shimadzu, Kyoto, Japan) with a frequency range of 700 to 4000 cm^−1^ and a resolution of 4 cm^−1^. AsRE (10 mg) was mixed with spectroscopic-grade potassium bromide powder and then pressed into 1.0 mm pellets and submitted to FTIR analyses.

### 2.5. In Vitro Antioxidant Activity

A single antioxidant assay is not sufficient to comprehensively assess the antioxidant capacity of extracts [23]. Therefore, in order to evaluate the activity of the extract at different stages of the oxidative cascade (initiation, propagation and termination), the antioxidant activity of the extract was evaluated by six different methods: total antioxidant capacity, reducing power, 1,1-Diphenyl-2-picrylhydrazyl (DPPH) radical scavenging test, cupric and ferric chelating, and hydroxyl radical scavenging. The IC_50_ (concentration required for 50% inhibition) value was determined from the plotted graph of reducing power, scavenging activity (DPPH and Hydroxyl radical) and metal chelating (Cupric chelating and ferric chelating) against the different concentrations of AsRE according to Equation (2).

IC_50_ value = (50-b/a) (2)

#### 2.5.1. Total Antioxidant Capacity (TAC)

The antioxidant capacity assay was assessed according to the method described by Costa et al. [24]. AsRE, together with a reagent solution (0.6 M sulfuric acid, 28 mM sodium phosphate and 4 mM ammonium molybdate), was incubated at 95 °C for 90 min. The mixture was cooled, and the absorbance measured at 695 nm against a blank. The antioxidant capacity was expressed as ascorbic acid equivalent.

#### 2.5.2. Reducing Power

The reducing power was evaluated as described previously [24]. Briefly, 4.0 mL of reaction mixture, containing different sample concentrations (0.05, 0.1, 0.25, 0.5, and 1.0 mg/mL) in phosphate buffer (0.2 M, pH 6.6), was incubated with potassium ferricyanide (1% *w*/*v*) at 50 °C for 20 min. The reaction was stopped by trichloroacetic acid solution (10% *w*/*v*). Afterwards, the solution was mixed with distilled water and ferric chloride solution (0.1% *w*/*v*). The absorbance was measured at 700 nm against a blank. The mixture was stirred, and the absorbance (700 nm) measured using a microplate reader (BioTek, Winooski, VT, USA). Results were expressed as the percentage of activity observed for 0.1 mg/mL (highest activity) ascorbic acid.

#### 2.5.3. 1,1-Diphenyl-2-Picrylhydrazyl (DPPH) Radical Scavenging Activity

AsRE’s ability to scavenge 1,1-Diphenyl-2-picrylhydrazyl (DPPH) free radicals was determined using the method described by Brand-Williams et al. [25]. Briefly, 1.0 mL of different concentrations of AsRE (0.25, 0.5, 1.0, 2.0, 4.0, 8.0 and 10.0 mg/mL) were added to 2.0 mL of 0.1 mM DPPH ethanolic solution. After 30 min at room temperature, the absorbances were measured at 517 nm. Butylated hydroxytoluene (BHT) (0.25, 0.5, 1.0, 2.0, 4.0, 8.0 and 10.0 mg/mL) was used as a positive control. The results were expressed as a percentage of radical scavenging according to Equation (3).

DPPH radical scavenging (%) = [1 − (As/A0)] × 100(3)

where As = absorbance of the sample; A0 = absorbance of the control.

#### 2.5.4. Cupric Chelating

The cupric chelating was evaluated as described previously [26]. Different concentrations of AsRE (0.1, 0.3, 0.5, 1.0, 1.5, and 2.0 mg/mL) were added to reagent solution (pyracotechol (4.0 mM) and cupric sulfate II pentahydrate (0.2 mM)) and measured at 632 nm. EDTA was used as a positive control (0.2–6.0 µg/mL). The results were expressed as a percentage of cupric chelation according to Equation (4).

Cupric chelating (%) = [(A0 − As)/A0] × 100(4)

where As = absorbance of the sample; A0 = absorbance of the control.

#### 2.5.5. Ferric Chelating

The ferric chelating effects of AsRE were evaluated as described previously by Decker and Welch [27]. Different concentrations (0.1, 0.3, 0.5, 1.0, 1.5, and 2.0 mg/mL) were added to reagent solution (FeCl_2_ 2.0 mM, Ferrozine 5.0 mM) and incubated at room temperature for 10 min at a wavelength of 562 nm. EDTA was used as a positive control (0.005–0.05 µg/mL). The results were expressed as a percentage of iron ion chelating according to Equation (5).

Ferric chelating (%) = [(A0 − As)/A0] × 100(5)

where As = absorbance of the sample; A0 = absorbance of the control.

#### 2.5.6. Hydroxyl Radical Scavenging Activity

The hydroxyl radical scavenging activity was evaluated using Fenton’s reaction as described previously [28]. Different concentrations of the AsRE (0.5, 1.0, 1.5 and 2.0 mg/mL) were added to reagent solution (10.0 mM iron sulphate, 10.0 mM EDTA, 2.0 mM sodium salicylate, 30% H_2_O_2_) and incubated at 37 °C for 1 h. Gallic acid (0.5, 1.0, 1.5 and 2.0 mg/mL) was used as positive control. The hydroxyl radical was investigated by monitoring absorbance at 510 nm. The results were expressed as a percentage of the scavenging of radicals according to Equation (6).

Hydroxyl radical scavenging (%) = [1 − (A0 − As)/(A0 − Ab)] × 100(6)

where As = absorbance of the sample; A0 = absorbance of the control; Ab = absorbance of the blank.

### 2.6. Caenorhabditis elegans Maintenance Strains and Extract Treatment

The N2 (wild type) strain of *C. elegans* was used, which was cultivated in the Nematode Growth Medium (NGM) with *Escherichia coli* OP50 and maintained at 20 °C [29]. Worms were synchronized by treating pregnant hermaphrodites with lysis solution (hypochlorite 2% and NaOH 10 mM) in order to obtain worms at the L1 stage. AsRE was added to the *E. coli* OP50 fluid at a final concentration of 1.0, 3.0 and 5.0 mg/mL. The fluid was added to the surface NGM [17].

### 2.7. Bacterial Growth Assay

The bacterial growth of *E. coli* OP*50* was assessed by optical density (OD) readings on the spectrophotometer (Biochrom Libra S22, Holliston, MA, USA). *E. coli* OP50 was cultivated into sterilized liquid medium with or without the different concentrations of AsRE (1.0, 3.0 and 5.0 mg/mL) in Luria–Bertani medium (LB). The nematodes were inoculated with the *E. coli* OP50 and separated into groups, respectively. The results expressed the relative levels. The initial values of optical density (OD600) to *E. coli* OP50 were compared at each point with the OD600 at the zero point of each condition. The samples were transferred to a rocking shaker at 37 °C (Marconi MA 420, Piracicaba, Brazil), and values measured once every 1 h up to 5 h.

### 2.8. Toxicity of Agave sisalana Extract Against C. elegans

#### 2.8.1. Effect of *A. sisalana* Agro-Industrial Residue Extract on Body Length

The worms synchronized at the L1 stage cultivated in NGM medium plates were separated into groups and submitted to treatment with AsRE at 1.0, 3.0 and 5.0 mg/mL for 48 h at 20 °C. In the control group, nematodes were cultured on the NGM with the *E. coli* OP50 fluid. In total, 30 worms from each group were photographed and body length measured using the ImageJ software (ImageJ 1.51j8-https://imagej.nih.gov/ij/). The experiment was performed in triplicate and results presented as mean area ± standard error of the mean (SEM).

#### 2.8.2. Effect of *Agave sisalana* Agro-Industrial Residue Extract on Hatchability

The effect of AsRE on the egg hatching of wild type *C. elegans* was evaluated for hatchability percentage. Embryos resistant to treatment with lysis solution were collected and added to the surface NGM medium in treatment groups at 1.0, 3.0 and 5.0 mg/mL (in *E. coli* OP50) and the control group only with *E. coli* OP50. After 24 h at 20 °C, hatchability percentage was calculated to a ratio of progeny number over brood size. Experiments were performed in triplicate and results presented as mean area ± standard error of the mean (SEM).

### 2.9. In Vivo Antioxidant Activity of Agave sisalana Agro-Industrial Residue Extract Using C. elegans as a Model Organism

#### 2.9.1. Intracellular Accumulation of Reactive Oxygen Species (ROS) in *C. elegans*

H_2_DCF-DA is converted into a non-fluorescent intermediate form that can later be oxidized by ROS to a fluorescent compound, DCF, in the intracellular medium. The fluorescence intensity of this oxidative derivative is correlated with the amount of intracellular ROS in a living organism. Wild type *C. elegans* from the L1 stage were treated with or without (control group) the different concentrations of AsRE (1.0, 3.0 and 5.0 mg/mL) for 48 h at 20 °C. Next, approximately 40 worms per group were transferred to a 96-well plate containing 50 μM H_2_DCFDA (2.7-dichlorofluorescein-diacetate) in PBS. Fluorescence quantification was performed on GloboMax^®^-mult detection system (Promega, Madison, WI, USA), with excitation at 490 nm and emission at 510–570 nm. Six readings were taken, with an interval of 30 min between each reading. The experiment was performed in three independent experiments, performed in triplicate and results presented as mean area ± standard error of the mean (SEM).

#### 2.9.2. Oxidative Stress Survival Assay

Wild type worms from the L1 stage were treated with AsRE at concentrations of 1.0, 3.0 and 5.0 mg/mL for 48 h at 20 °C. Subsequently, 50 nematodes per group were transferred to plates with NGM solid medium containing 10 mM tert-butyl hydroperoxide (TBHP). The fraction of surviving nematodes was evaluated for up to 33 h using a stereoscopic microscope by probing the nematodes. This assay was repeated three times with a total number of 150 worms/group and results presented as mean area ± standard error of the mean (SEM).

#### 2.9.3. Expression Levels of the Oxidative Resistance Relative Gene

CL2166 mutant worms carrying an inducible green fluorescence protein (GFP) reporter for *gst-4* (*gst-4::GFP*) were maintained on NGM plates with or without (control) different concentrations of AsRE (1.0, 3.0 and 5.0 mg/mL) for 48 h. The gene expression was determined by fluorescence microscopy (Olympus BX51, BR). The fluorescence intensity was quantified using the ImageJ software (ImageJ 1.51 j8).

### 2.10. Statistical Analysis

All experiments were performed three times. Statistical analysis was performed using GraphPad Prism 8.0.2 (https://www.graphpad.com/scientificsoftware/prism/). Student’s t test was used to compare pairs of groups, as well as a one-way ANOVA followed by Tukey’s post test. For the hydroxyl radical scavenging test, a two-way way ANOVA followed by Sidak’s multiple comparisons was used. Survival curves were analyzed by the log-rank (Mantel–Cox) test.

## 3. Results

### 3.1. Chemical Characterization

**Figure 1:**
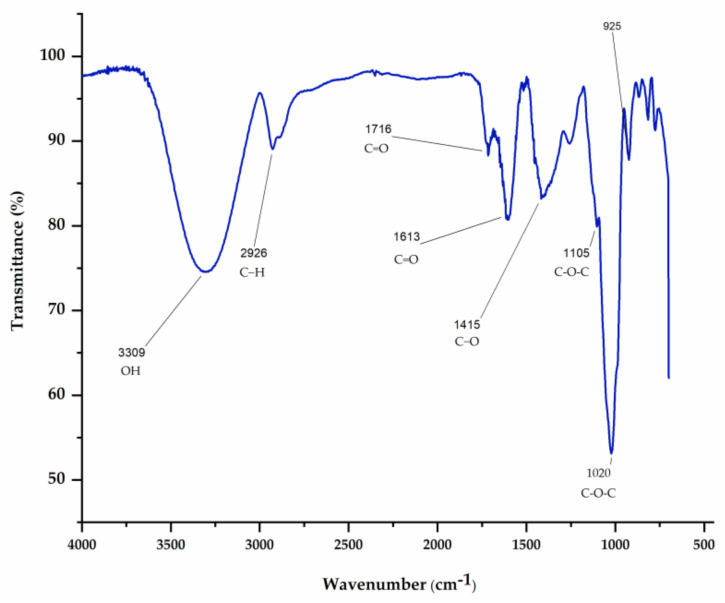
Infrared spectra of *A. sisalana* agro-industrial residue extract (AsRE); bands at: 3309 attributed to a hydroxyl stretching vibration [23]; 2926 attributed to the C−H asymmetric stretching vibration [30]; 1020 attributed to C−O stretching vibrations [31] and 1105 and 1020 attributed to C−O−C [32]; 1716 attributed to C=O [33]; 1415 cm^−1^ and 1613 cm^−1^ corresponded to C−O [23] and the free carboxyl group, respectively [34,35].

Saponins and polyphenols are the most widely studied classes of the *Agave* genus. After the extractive process, the yield for AsRE was 7.08%. The results obtained showed that saponins comprise 18.01% ± 1.81 of AsRE while the concentration of total flavonoids is 0.92 ± 0.26 µg EQ/mg. The infrared spectra of AsRE displayed some characteristic bands compatible with polysaccharides, saponins and phenolics (Figure 1).

### 3.2. In Vitro Antioxidant Activity

**Figure 2:**
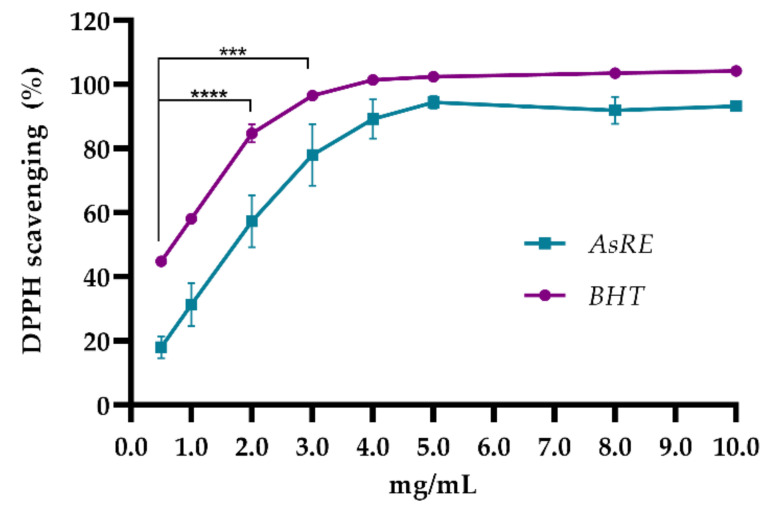
DPPH radical scavenging assay; AsRE = *A. sisalana* agro-industrial residue extract; *** *p* < 0.001 and **** *p* < 0.0001, by two-way ANOVA, by one-way ANOVA, followed by a Tukey’s post-test. The assay was carried out in three independent experiments and performed in triplicate. The results were expressed as mean ± DP.

The total antioxidant capacity test (TAC) showed a high activity for AsRE (91.75 mg Eq AA/g). The reducing power assay showed AsRE (1.0 mg/mL) with an activity of 49.13% ± 2.03 in comparison to ascorbic acid (100 µg). When AsRE was assessed for its ability to scavenge DPPH radical scavenging, it showed that it was able to scavenge the radicals in a concentration-dependent manner up to a concentration of 5.0 mg/mL with maximum values of 94.40% ± 1.94 (Figure 2). Starting at the concentration of 4.0 mg/mL and higher concentrations, there is no significant difference between AsRE and the positive control used.

**Figure 3:**
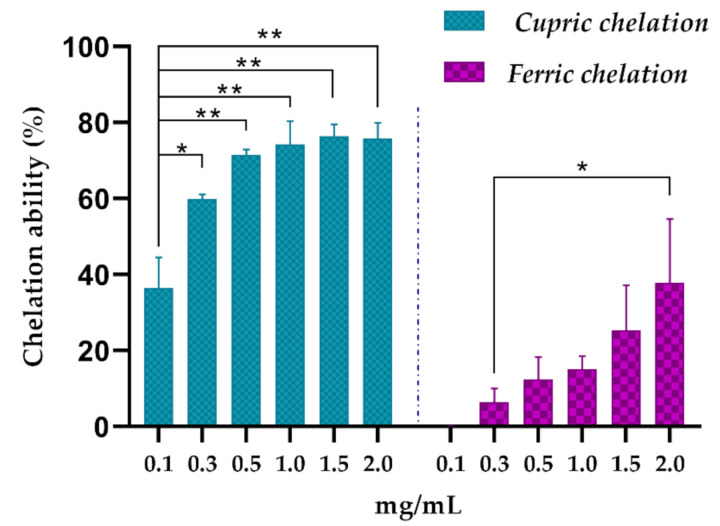
Metal chelating capacity of *A. sisalana* agro-industrial residue extract. Cupric and ferric chelating activity. * *p* < 0.05 and ** *p* < 0.01 by one-way ANOVA. The assay was carried out in three independent experiments, performed in triplicate. The results were expressed as mean ± DP. For cupric chelating, the positive control used (EDTA) reached maximum inhibition from the concentration at 0.03 µg. For ferric chelating, the positive control used (EDTA) reached maximum inhibition from the concentration at 5.0 µg.

In relation to the metal chelating capacity, the extract showed a high cupric chelating power (70% at 0.5 mg/mL). Furthermore, for ferric chelating activity, the extract (1.0 mg/mL) showed 15.10% ± 3.44 chelating activity (Figure 3).

**Figure 4:**
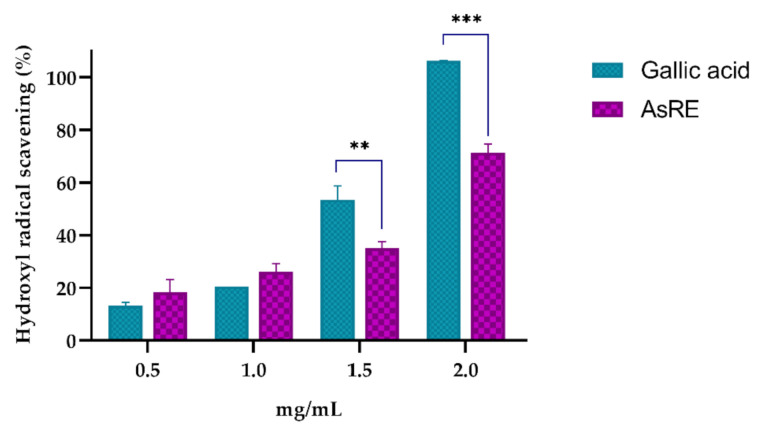
Antioxidant potential of *A. sisalana* agro-industrial residue extract. AsRE = *A. sisalana* agro-industrial residue extract; ** *p* < 0.01 and *** *p* < 0.001 by two-way ANOVA, followed by a Sidak’s multiple comparisons test. The assay was carried out in three independent experiments, performed in triplicate. The results were expressed as mean ± DP.

AsRE demonstrated hydroxyl radical scavenging in a concentration-dependent manner, reaching 71.38% ± 3.25 at 2.0 mg/mL (Figure 4).

The results presented demonstrate the potential of the extract as an antioxidant raw material with possible intrinsic performances in different phases of the oxidative cascade. The IC_50_ values obtained for antioxidant tests can be seen in Table 1.

### 3.3. Bacterial Growth Assay

Before the in vivo antioxidant tests, the effect of the extract on the growth of *E. coli* OP50 bacteria, used as a food source, was evaluated. The toxicity against *E. coli* OP50 can lead to an increase in the lifespan of *C. elegans* [17]. The AsRE (1.0, 3.0 and 5.0 mg/mL) did not exhibit antibacterial activity against *E. coli* OP50.

### 3.4. Toxicity of Agave sisalana Extract Against C. elegans

**Figure 5:**
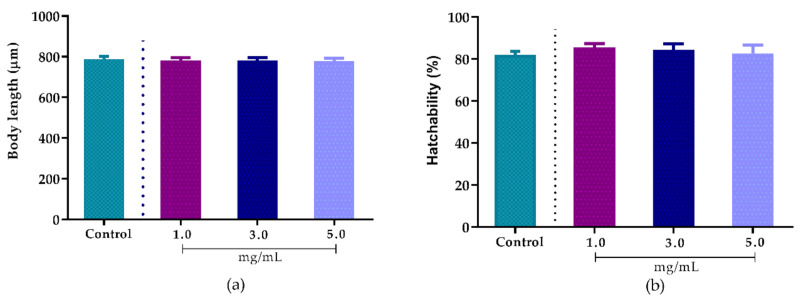
Effect of *Agave sisalana* agro-industrial residue extract on worm development. (**a**) Body length after treatments with 1.0, 3.0 and 5.0 mg/mL of extract; (**b**) Hatchability percentage after treatments with 1.0, 3.0 and 5.0 mg/mL of extract.

After 48 h of exposure to different concentrations (1.0, 3.0 and 5.0 mg/mL) of the extract, no significant changes were observed in terms of body length (Figure 5a) or hatchability (Figure 5b), indicating that the fractions do not present toxicity to the nematodes.

### 3.5. In Vivo Antioidant Activity

#### 3.5.1. Intracellular Accumulation of ROS in *C. elegans*

**Figure 6:**
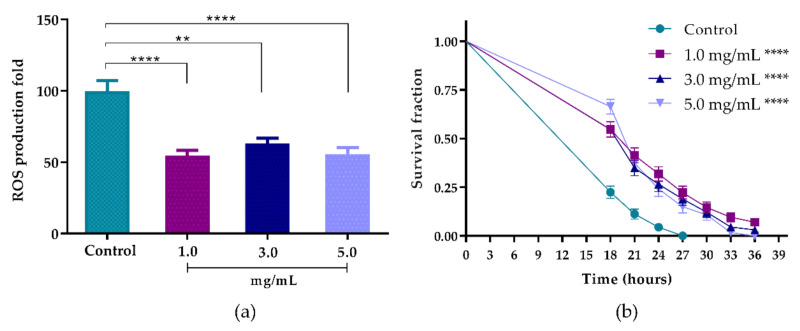
Intracellular levels of ROS and survival rate curve of wild-type N2 worms treated with AsRE. (**a**) ROS production levels after treatment with 1.0, 3.0 and 5.0 mg/mL of AsRE. (**b**) Survival fraction curves of *C. elegans* under stress conditions after treatment with 1.0, 3.0 and 5.0 mg/mL of AsRE; ** *p* < 0.01 compared control with 3.0 mg of treatment; **** *p* < 0.0001 compared control with 1.0 and 3.0 mg of treatment; by one-way ANOVA, followed by a Tukey’s post-test; (**b**) Survival fraction after treatment with 1.0, 3.0 and 5.0 mg/mL of extract. Survival was measured every 3 h at 25 °C; **** *p* < 0.0001, compared to the control with worms treated by log-rank (Mantel–Cox) test.

Nematodes treated with 1.0, 3.0 and 5.0 mg/mL of the extract showed a reduction in intracellular ROS levels of 45.18% (*p* < 0.0001), 36.84% (*p* < 0.01) and 44.19% (*p* < 0.0001), respectively (Figure 6a). However, no statistical differences were observed among the AsRE at different concentrations.

#### 3.5.2. Oxidative Stress Survival Assay

The reduction in intracellular levels of ROS enabled us to evaluate the ability of the extract to increase the survival fraction of worms against the acute stress induced (TBHP). The extract promoted a significant change to the right in the survival curve under acute oxidative stress induced at the concentrations evaluated when compared to the control (*p* < 0.0001) (Figure 6b). The increase in average life span when compared to the control group (*p* < 0.0001) can be seen in Table 2. The OS process leads to cumulative damage to biomolecules that promote the aging process and consequently the survival time of *C. elegans* [13]. Therefore, the extension of the lifespan is strongly associated with enhanced resistance to stress [15]. Thus, we suggest that there is an increase in the survival fraction with a reduction in the ROS levels promoted by the extract.

#### 3.5.3. Effect of AsRE on the Expression Levels of the Oxidative Stress Relative Gene (*gst-4::GFP*)

**Figure 7:**
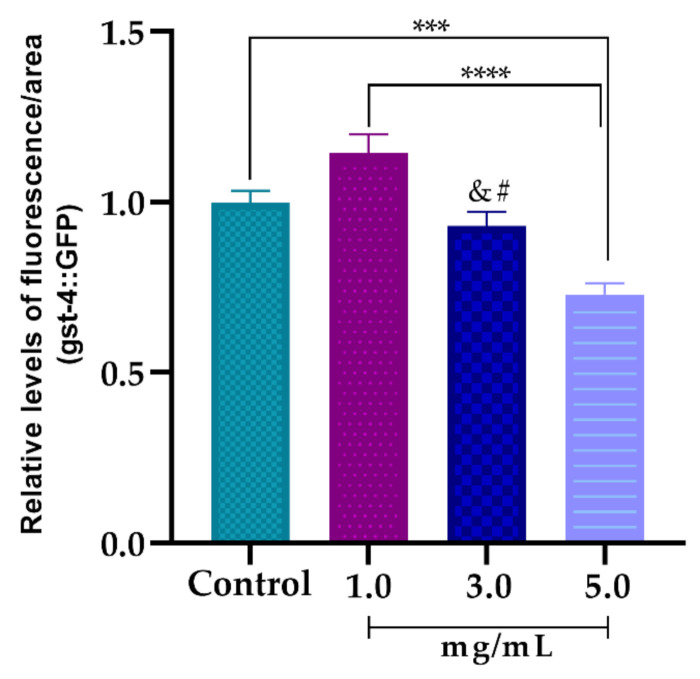
Effect of *A. sisalana* agro-industrial residue extract (AsRE) on the expression levels of the oxidative stress relative gene *gst-4::GFP*; *** *p* < 0.001 compared control with 5.0 mg/mL of treatment; **** *p* < 0.0001 compared treatment of 1.0 mg and 5.0 mg/mL; # = ** *p* < 0.01 compared treatment of 3.0 and 1.0 mg/mL; & = * *p* < 0.05 compared treatment of 3.0 and 5.0 mg/mL; by one-way ANOVA, followed by a Tukey’s post-test. The assay was performed in duplicate and the results expressed as mean ± DP.

The CL2166 worms were previously treated with 1.0, 3.0 and 5.0 mg/mL of AsRE to assess their ability to modulate the expression of genes related to oxidative stress resistance (*gst-4::GFP*). A dose-dependent profile was observed in the negative expression of the *gst-4::GFP* gene (Figure 7). However, when compared to the control, only the concentration of 5.0 mg/mL showed a significant reduction in the expression of *gst-4::GFP*, reducing the fluorescence intensity by 27.04% ± 0.04.

## 4. Discussion

The search for new molecules with biological activity has been intensified in studies with plant extracts due to their diverse and complex chemical constitution. In a previous study, our group showed that *A. sisalana* extract contained polyphenols (2.53%) and sugars (65%) [10]. Now, in this present study, to advance the chemical characterization of this extract, the total saponins and total flavonoids were analyzed. The infrared analyses also indicated the presence of sugar (OH, C-H, C-O-C, C-O, C=O) [23,30,34,35], saponins (CO, C-O-C, C=O) [32,33], and flavonoids [36] in AsRE.

It is interesting to highlight that among the phenolic compounds of the genus *Agave*, flavonoids, homoisoflavonoids and phenolic acids were the most common phenolic compounds found [7]. Therefore, we hypothesize that the low amount of total flavonoids could indicate a richness of phenolic acid in the extract. Furthermore, the presence of saponin characterization data was an expected result regarding the historical importance and occurrence of these metabolites in the genus. Taken together, these features are compatible with the expected chemical profile of *A. sisalana* hydroethanolic extract and these biomolecules demonstrated a strong correlation with biological activities, such as antioxidant activity [5,8].

In particular, molecules with antioxidant capacity are used in order to combat the damage caused by oxidative stress and to prevent aging [37,38]. Many studies with extracts and their components have demonstrated the correlation between antioxidant activity and primary and secondary metabolites of plants such as polyphenols [39,40], saponins [8] and polysaccharides [41,42,43,44].

Phenolic compounds are constituted of one or more aromatic rings containing variable hydroxyl groups, and are, therefore, potentially capable of stabilizing reactive species by forming resonance-stabilized phenoxy radicals [45]. The activity of saponins, on the other hand, has been related to their glycosylated C-3 hydroxyl group [8], while polysaccharides can donate atoms and stop chain reactions by turning radicals into stable non-harmful molecules. According to Huang et al. [44], the carboxyl and carbonyl groups, present in polysaccharides, decrease the dissociation energy of the O-H bond, which results in increased proton release, potentiating the antioxidant activity of these compounds.

The antioxidant activity of AsRE was evaluated in three stages: initiation (Total Antioxidant Capacity, power reducing and DPPH radical scavenging), propagation (cupric and ferric chelating), and termination (hydroxyl radical scavenging) [46].

The total antioxidant capacity test showed an activity for the AsRE of 91.75 mg EqAA/g, which is considered high antioxidant activity [24]. Moreover, AsRE showed high reducing power when compared with positive control ascorbic acid (49.13% ± 2.03). A previous study using isolated polysaccharides of agro-industrial *A. sisalana* showed a power reduction of 37% compared to ascorbic acid [5]. Reducing properties are generally associated with the presence of biomolecules, which exhibit antioxidant activity by scavenging effect on the reactive species [17], donating hydrogen atoms to break chain reactions [5].

AsRE showed high DPPH radical scavenging activity when compared to the positive control (Figure 2). Polysaccharides isolated from this material demonstrated the ability to interact with oxidative processes acting as an antioxidant agent [5,11]. Furthermore, Ribeiro et al. [8] pointed out a more significant activity for the extract when compared to the isolated compounds. These authors highlighted the low ratio of saponins isolated from the leaves of *A. sisalana* with the elimination of DPPH radicals (IC_50_ > 10.0 mg/mL), while the extract containing saponins and polyphenols showed results (IC_50_ = 1.4 mg/mL) similar to those observed for AsRE (IC_50_ = 1.44 mg/mL). Therefore, it is possible to imply that the diversified chemical composition of this plant, when compared to isolated molecules, may favor a greater antioxidant potential. The significant results of the total antioxidant capacity and reducing power in vitro indicate that AsRE has the ability to interact with the systems and give electrons to minimize the attack of radicals [47], which correlates with the ability to eliminate radicals [5].

AsRE demonstrated chelating activity of cupric and iron (Figure 3). Several studies showed plant extracts with iron and/or cupric chelating activity. These authors relate this chelating activity to the presence of molecules such as polyphenols [48] and polysaccharides [5,24,42,49]. The evaluation of the metal chelating potential is important due to transition metals, free of redox activity, such as Cu^2^+ and Fe^2^+, which can be extremely pro-oxidant, meaning that they can react with hydrogen peroxide and catalyze the formation of hydroxyl radical through the Fenton reaction [37]. Therefore, when AsRE acts as a metal chelator, it is indirectly combating the damage caused by the hydroxyl radical. However, there are two types of antioxidant mechanism against the hydroxyl radical: one suppresses the generation of the hydroxyl radical and the other scavenges the hydroxyl radicals generated [24]. Due to this, the potential of AsRE for radical hydroxyl scavenging was also evaluated (Figure 4). The scavenging ability of AsRE for hydroxyl radicals increased with the concentration within the range of 0.5 e 2.0 mg/mL. The rate that AsRE and gallic acid scavenged hydroxyl radicals at 2.0 mg/mL was 71.38% and 100%, respectively. Zhang et al. [5] showed the scavenging effect on the hydroxyl radical by polysaccharides of *A. sisalana* residue. However, the activity found for these metabolics was around 33% at 4.0 mg/mL. Biomolecules and extracts of other sources also demonstrated activity for scavenging hydroxyl radicals. Cheng et al. [50] showed the activity of flavonoids from *Carex meyeriana* on scavenging the hydroxyl radical. *Melocactus zehntneri* ethanolic extract containing phenolic compounds, saponins and polysaccharides showed 100% hydroxyl scavenging activity for polar extract (using 100 µg/mL), whereas the non-polar extracts showed an activity of around 20% [46]. Therefore, AsRE exerted their antioxidant activity in three stages: initiation, propagation and termination.

Considering that AsRE showed excellent in vitro antioxidant activity, the nematode *C. elegans* was used to evaluate the in vivo antioxidant potential of AsRE. In addition, after the results obtained in the in vitro tests and knowing that high concentrations are used in tests with these worms due to the presence of a thick cuticle [18], the concentrations 1.0, 3.0 and 5.0 mg/mL were used in the tests in vivo.

In first step, bacterial Growth of *E. coli* OP50 was evaluated. The worms feed on *E. coli* OP50 and the inhibition of this bacteria can lead to an increase in the lifespan of *C. elegans* [17]. Consequently, if the extract caused the death of the bacteria or growth inhibition, the worms should be fed with the dead bacteria so that the increase in the lifespan does not interfere with the results of the antioxidant tests performed with this model. However, AsRE did not interfere with the bacterial growth of *E. coli* OP50, so it was not necessary to feed them with dead bacteria. The non-toxicity of AsRE corroborates the results obtained by Santos et al. [51], which showed that hydroethanolic extracts from *A. sisalana* leaves and residue have no relevant activity against the bacteria tested. Similar results were observed by Ribeiro et al. [8] when evaluating saponins from *A. sisalana* to *E. coli*.

An anthelmintic effect, in vitro, against goat gastrointestinal nematodes of *A. sisalana* agro-industrial residue has been demonstrated by Domingues et al. [52]. Therefore, the ovicidal and larvicidal in vitro effect of *A. sisalana* was shown by Botura et al. [53] using gastrointestinal nematodes of goats. However, AsRE did not affect the development of *C. elegans* nematodes.

The results for the antioxidant capacity (Figure 6) obtained with the *C. elegans* model reinforce the results observed in vitro. AsRE was able to act as an antioxidant by reducing the intracellular levels of ROS in wild-type worms and by increasing survival rates after they had been induced to strong oxidative stress. ROS are highly reactive, usually produced by the cellular aerobic metabolism that can increase dramatically when organisms are exposed to different stress conditions [54].

The *C. elegans* results involving the reduction in intracellular levels of ROS are commonly associated with an increase in the rate of survival of these animals, when induced to stress. Different studies have shown plant extracts with antioxidant activity capable of reducing ROS levels and increasing the *C. elegans* survival rate. Azevedo et al. [16] demonstrated that the aqueous extract of *Uncaria tomentosa* leaves was also able to reduce ROS levels and increase the survival rate of *C. elegans*. *Panax notoginseng* ethanol extracts also increased lifespan and protected *C. elegans* against oxidative stress. Similar results were observed by Ribeiro et al. [18] when using ethanol extract from *Myrciaria tenella* leaves. Moreover, a correlation between stress resistance and lifespan extension has been attributed to polyphenols [13,55] and saponins [56].

However, antioxidants can act directly to reduce ROS levels [16] or to increase resistance to oxidative stress, inducing the transcription of cytoprotective proteins [57]. To understand the mechanisms by which AsRE can act against oxidative stress, glutathione S-transferase (*gst-4*) gene expression levels were evaluated. *gst-4* is a phase II detoxification enzyme that catalyzes the conjugation of several exogenous and endogenous electrophiles with the reduced form of glutathione, resulting in the detoxification of ROS [56].

AsRE promoted a reduction in expression of gst-4 levels in a dose-dependent manner (Figure 7). The capacity to suppress expression of *gst-4* is widely interpreted as proof of their ability to reduce oxidative stress in *C. elegans* [16,56]. Accordingly, it could be suggested that the reduction in intracellular levels of ROS associated with increased resistance to stress and downregulation of *gst-4::GFP* expression is related to the direct activity of the AsRE against ROS.

AsRE was able to reduce the production of intracellular ROS and increase the mean lifespan of wild-type *C. elegans* under stress conditions. Since oxidative stress plays a role in many diseases and contributes to aging [12], AsRE may lead to the production of a new raw material against oxidative stress and aging in the near future. This agro-industrial residue can be considered a potential renewable, sustainable, and environmentally friendly source. Hence, it is possible to reincorporate it into the industry in new production processes, which can also generate extra income and contribute to socioeconomic development in semi-arid regions.

## 5. Conclusions

*A. sisalana* agro-industrial residue extract has a diverse chemical composition and proven antioxidant activity. In this study, the antioxidant evaluation, in vitro and in vivo, using the *C. elegans* organism model showed evidence that the extract can efficiently eliminate reactive species in vitro and in vivo, reducing endogenous levels of reactive species and promoting an increased survival rate of *C. elegans* under stress, acting directly on the elimination of reactive species. However, new tests should be carried out to explain the relationship between in vivo and in vitro assays. *A. sisalana* agro-industrial residue extract can be considered a potential antioxidant raw material for different applications in the food, cosmetic and pharmaceutical industries. We can also point out as advantages of this studied extract the ease and availability of obtaining biomass and the fact that the production process of the extract is industrially scalable, fundamental conditions for it to become commercial. This enables the prospect of introducing to the agroindustry a renewable process that promotes sustainability and socioeconomic development and brings benefits to current and future generations.

## Figures and Tables

**Table 1:** IC_50_ values of *Agave sisalana* agro-industrial residue extract obtained from antioxidant tests.

Antioxidant Tests	IC_5O_ Value (mg/mL)
Reducing power	1.02
DPPH radical scavenging	1.44
Cupric chelating	0.16
Ferric chelating	2.76
Hydroxyl radical scavenging	1.61

**Table 2:** Evaluation of resistance to oxidative stress in nematodes treated with the extract.

	Concentrations (mg/mL)	Mean Survival (hours ± SEM)	% Mean Survival Time Variation vs. Untreated	*p* Value (Long Rank) Extract vs. Untreated	n ^1^
**Control**		19.14 ± 0.19	-	-	165 (3)
**As** **RE**	1.0	23.23 ± 0.49	21.37	<0.0001	159 (3)
	3.0	22.55 ± 0.47	17.82	<0.0001	154 (3)
	5.0	22.66 ± 0.44	18.39	<0.0001	152 (3)

Where: n ^1^ = number of animals used in experiment; AsRE = *A. sisalana* agro-industrial residue extract.
